# Locking compression plate fixation of femoral intertrochanteric fractures in patients with preexisting proximal femoral deformity: a retrospective study

**DOI:** 10.1186/s13018-021-02430-5

**Published:** 2021-04-29

**Authors:** Shan Fan, Mingming Yin, Yibo Xu, Cheng Ren, Teng Ma, Yao Lu, Ming Li, Zhong Li, Kun Zhang

**Affiliations:** 1 Department of Surgery and Anesthesiology II, Xi’an Honghui Hospital, School of Medicine, Xi’an Jiaotong University, Xi’an, 710054 P. R. China; 2 Department of Burn and Microsurgery, The People’s Hospital of Ankang, Ankang, Shaanxi Province 725000 P.R. China; 3 Department of Orthopedics Trauma, Xi’an Honghui Hospital, School of Medicine, Xi’an Jiaotong University, Xi’an, 710054 P.R. China

**Keywords:** Intertrochanteric fracture, Proximal femoral deformity, Fracture fixation, Locking compression plate

## Abstract

**Background:**

To investigate the clinical efficacy of locking compression plate fixation for the treatment of femoral intertrochanteric fractures in patients with preexisting proximal femoral deformity.

**Methods:**

A retrospective analysis was conducted on 37 patients with femoral intertrochanteric fractures combined with preexisting proximal femoral deformity between January 2013 and July 2019. The patients included 24 males and 13 females aged from 23 to 69 years old, with an average age of 47.5 years. The preexisting proximal femoral deformities resulted from poliomyelitis sequela, proximal femoral fibrous dysplasia, malunion and implant failure combined with coxa vara after intramedullary nailing fixation. There were 6 cases of 31-A2.1, 6 cases of 31-A2.2, 20 cases of 31-A3.1, and 5 cases of 31-A3.2, determined based on the AO classification of intertrochanteric fractures. All fractures were managed through open reduction and locking plate fixation. The hip disability and osteoarthritis outcome score (HOOS) was used to assess hip function before injury and at the last postoperative follow-up. The short form 36 (SF-36) Health Survey Questionnaire was used to assess quality of life.

**Results:**

Thirty-seven patients were followed up for 12 to 27 months (average, 20.7 months). All patients achieved bone healing within 5.1 months on average (range, 3 to 6 months). Postoperative complications included deep vein thrombosis in three patients, bedsores in one and delayed union in one patient. No other complications, such as surgical site infection, fat embolism, nonunion and re-fracture, were presented. There was no significant difference in the HOOS scores and the SF-36 Health Questionnaire outcomes at pre-injury and at the last postoperative follow-up (*p* > 0.05).

**Conclusions:**

It is difficult to perform intramedullary fixation of femoral intertrochanteric fractures in patients with preexisting proximal femoral deformity, while locking compression plate fixation is a simple and effective method of treatment.

## Introduction

The incidence of intertrochanteric fractures has been increasing every year [1, 2]. Due to the development of minimally invasive surgery methods, intramedullary fixation has been recently used to treat femoral intertrochanteric fractures. On the other hand, proximal femoral deformity in adults can be induced by a wide variety of primary diseases, including poliomyelitis sequela, proximal femoral fibrous dysplasia, malunion after fracture, internal fixation implants failure, and the residual deformities from previous osteotomy [3–5], which may cause unpredictable pathological and biomechanical changes of the proximal femur and femoral osteotomy. Therefore, total hip arthroplasty for femoral reconstruction and fixation are conventional treatment options used for patients without proximal femoral fractures [6].

However, when intertrochanteric fracture and proximal femoral deformity are presented simultaneously, due to the unpredictable fracture pattern, complex proximal femoral geometry and deformity morphology [7], more accurate clinical decision-making, higher technical requirements for fracture reduction, and a more sophisticated postoperative rehabilitation experience are essential prerequisites for excellent and good clinical outcomes of the patients, and is a substantial challenge for intramedullary fixation or total hip arthroplasty and osteotomy for the treatment of femoral intertrochanteric fractures in patients with preexisting proximal femoral deformities. Orthopedic surgeons are faced with the dilemma of performing a simple and effective internal fixation procedure on these patients to restore optimal hip function. Miclau et al. [8] reported that after the installation of locking sleeve, reshaping of the locking compression plate in an appropriate range would neither seriously damage the thread of the nail hole nor affect the locking strength between the locking screw and the plate. Therefore, the locking plate-screw-fracture fragment could be completely and stably integrated so that screw loosening and bone plate breakage could be avoided. Therefore, we retrospectively analyzed the data on femoral intertrochanteric fractures in 37 patients with preexisting proximal femoral deformity between January 2013 to July 2019. All fractures were fixed with locking compression plates and achieved excellent and good clinical outcomes.

## Materials and methods

### Inclusion and exclusion criteria

Inclusion criteria: (1) patients aged over 18 years old; (2) patients with fresh fractures; (3) X-ray film and CT scan demonstrating femoral intertrochanteric fractures and preexisting proximal femoral deformity; (4) no knee dislocation, no vascular or nerve injury; (5) complete clinical demographic and follow-up data. Exclusion criteria: (1) patients with open fractures; (2) patients who refused surgery; (3) incomplete clinical demographic and follow-up data.

### Study design

A single-center, case series, retrospective study was conducted at the Lower Limb Surgery Ward of the Traumatic Orthopedic Department, Xi’an Honghui Hospital from January 2013 to July 2019. All eligible data including age, gender, mechanism of injury, AO classification, days before operation, complications, time of bone healing, HOOS, and SF-36 scores were collected and reviewed. Thirty-seven patients, including 24 males and 13 females aged from 23 to 69 years old with a mean age of 47.5 years, were enrolled in this study. According to the distribution of the mechanism of injury, 31 cases resulted from accidental falling from a standing height and 6 cases from a road traffic accident. The causes of the preexisting proximal femoral deformities, included 8 cases of proximal femoral fibrous dysplasia, 9 cases of poliomyelitis sequela, 18 cases of malunion, and 2 cases of internal fixation implant failure combined with coxa vara after intramedullary nailing fixation. There were 6 cases of 31-A2.1, 6 cases of 31-A2.2, 20 cases of 31-A3.1, 5 cases of 31-A3.2 based on AO classification of intertrochanteric fractures. All patients or their families provided written informed consent for participation before surgery and the study was approved by the Ethics Committee of Xi’an Honghui Hospital.

### Surgical procedure

The patients were placed under epidural anesthesia or general anesthesia in the supine position on an orthopedic surgical table. The proximal femoral lateral approach was performed after routine disinfection and toweling with an average length of 15 cm ranged from 12 to 18 cm depending on the pattern of the fracture. The skin, subcutaneous tissue, and fascia were then bluntly separated from the lateral femoral muscle layer-by-layer to expose the fracture site. The lateral side of the proximal femur was measured using a testing template after the fracture was successfully restored, which was then used as a reference to reshape the locking plate. The plate was placed on the lateral side of the proximal femur and locking screws were placed into each appropriate hole according to the position of the fracture line. For patients with poliomyelitis sequela, the main challenge was the presence of an abnormal femoral neck angle and anteversion angle and an extremely narrow medullary cavity, which caused difficulty in achieving effective fixation using a 4.5-mm bone plate. Under these circumstances, we resorted to epiphyseal plate fixation so that the proximal end could be fixed with multiple screws and at different angles. Patients with proximal femoral fibrous dysplasia were treated by completely removing lesions in the femur, followed by reshaping of the proximal femoral locking compression plate or reverse distal femoral locking compression plate [9], which was then used for fixation after implantation of the autologous iliac bone combined with allogeneic bone. For patients with malunion, the fracture was reduced under direct vision based on the location of the fracture and fixed with reverse distal femoral locking compression plate. Patients with implant breakage and coxa vara after proximal femoral nail fixation were fixed with reverse femoral LISS bone plate after removal of the original failure internal fixation plate. A 3D model was printed prior to surgery and was used for bone plate reshaping, decision-making of plate length, and the number of screws. The reduction and internal fixation of the fractures were evaluated using intraoperative C-arm fluoroscopy. Once satisfactory reduction was achieved, irrigation and suction drainage were performed and the incision was subsequently sutured.

### Postoperative management and follow-up

After cold compress for 24 h and routine second-generation cephalosporin antibiotics were administered postoperatively for 48 h to prevent infection, and the drainage tube was removed. Comorbidity diseases, such as osteoporosis, were treated using alendronate and vitamin D3. Physical prevention (quadriceps muscle contraction and relaxation exercise, and CPM training) and thrombolytics or anticoagulant therapy were administered after surgery to prevent deep vein thrombosis of the lower extremities. If the patient presented with deep vein thrombosis, different methods of treatment were provided according to the location of the thrombosis. The inferior vena cava filter implantation was performed if thrombosis located proximal to the level of the popliteal vein to decrease the risk of pulmonary embolism, otherwise only low molecular weight heparin was administered. X-rays of the anteroposterior and lateral femurs were taken after the postoperative removal of the drainage tube to investigate reduction and implant location, and the wound sutures were removed after 2 weeks. Follow-up took place once a month for the first 3 months after surgery, then every 3 to 6 months after the first 3 follow-ups visits of the surgery, and finally every 6 to 12 months after 1 year of surgery. The presence of a callus formation observed through X-ray examination implied that the patient was ready for partial weight-bearing activity, while full weight-bearing depended on the condition of the fracture healing.

### Observation indexes

Various indexes, including days before surgery, bone formation condition, complications (internal fixation loosening, fracture, nonunion, and deep infection), and fracture healing time, were assessed through the hip disability and osteoarthritis score (HOOS) after fixation with the locking compression plate, which is generally adopted for total hip arthroplasty for hip osteoarthritis. However, it shows good discriminability in evaluating the outcomes of hip and femoral fractures [10, 11]. Hip function was evaluated based on 5 aspects: pain, symptoms, activity limitations-daily living, sport and recreation function, and quality of life [12]. When the patient was first admitted to hospital, they were asked to self-evaluate the quality of life less than 6 months before injury using the SF-36 Health Questionnaire [13], and at the last follow-up, the postoperative quality of life. The 8 SF-36 subscales included physiological function, role-physical, bodily pain, general health, vitality, social function, role-emotional, and mental health. A higher score indicated lower functional damage and a better quality of life.

### Statistical analysis

Statistical analysis was performed using SPSS 24.0 statistical software (IBM, USA). A Shapiro-Wilk test for normality was conducted on all continuous data, and continuous data with a normal distribution were described as mean ± standard deviation, whereas categorical data was described in several cases (percentages). The hip scores of the HOOS and the SF-36 Health Questionnaire were expressed as mean ± standard deviation. The HOOS and the SF-36 Health Questionnaire scores before injury and at the last follow-up were compared using Student’s *t* test and the Mann-Whitney *U* test for those without normal distribution. The *χ*2 test was performed for categorical variables. A *p* value of < 0.05 was selected as the threshold of statistical significance.

## Results

All 37 patients were followed-up for 12 to 27 months, with an average follow-up duration of 20.7 months. The number of days before surgery varied from 2 to 6 days, with an average of 4.0 days. In our study, the wounds healed perfectly, as expected, and the sutures were removed 2 weeks after surgery. Postoperative complications included deep vein thrombosis in three patients, bedsores in one and delayed union in one patient. No other complications, such as surgical site infection, pneumonia, fat embolism, nonunion, or re-fracture were presented by any of the patients. Three months after surgery, significant amounts of callus continuously formed at the fracture site and the locking compression plate remained in the correct position. At 6 months after surgery, the position of the locking compression plate was still correct and intact without nail retraction, loosening, or breaking. The time of bone healing ranged from 3 to 6 months (average, 5.1 months).

**Fig. 1:**
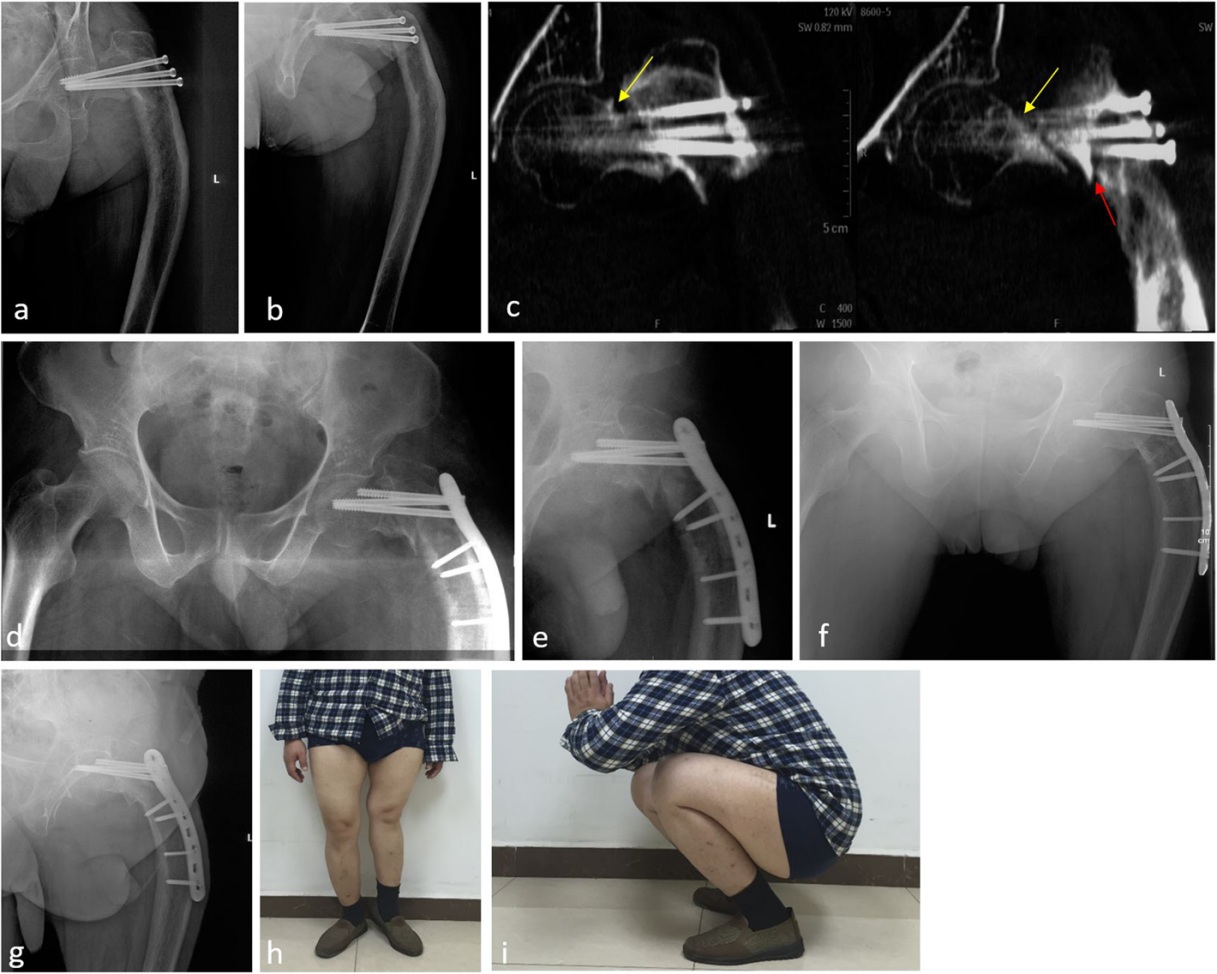
The patient was a 41-year-old blind male who accidently fell from a height, which caused left hip pain and limited activity. Two months prior, he had presented with ipsilateral femoral neck fracture due to falling from a standing height and was treated with closed reduction and cannulated screws fixation. Additionally, 34 years ago, the left thigh was subjected to a proximal femoral fracture, which was conservatively treated. A preoperative anteroposterior X-ray (**a**) and a lateral X-ray (**b**) demonstrated a left femoral intertrochanteric fracture, cannulated screws fixation for previous femoral neck fracture, and a proximal femoral angulation deformity due to the injury 34 years ago. CT radiographs (**c**) indicated a clear fracture line of the left femoral neck fracture (yellow arrow) and the ipsilateral femoral intertrochanteric fracture (red arrow). The cannulated screws were removed and the reshaped proximal femoral locking compression plate was used for fixation. Postoperative X-ray radiographs of the anteroposterior (**d**) and lateral view (**e**) 2 days after surgery. X-ray radiographs of the anteroposterior (**f**) and lateral view (**g**) 2 years after surgery indicated fracture union. The general functional outcomes at the 2-year follow-up (**h**, **i**)

**Fig. 2:**
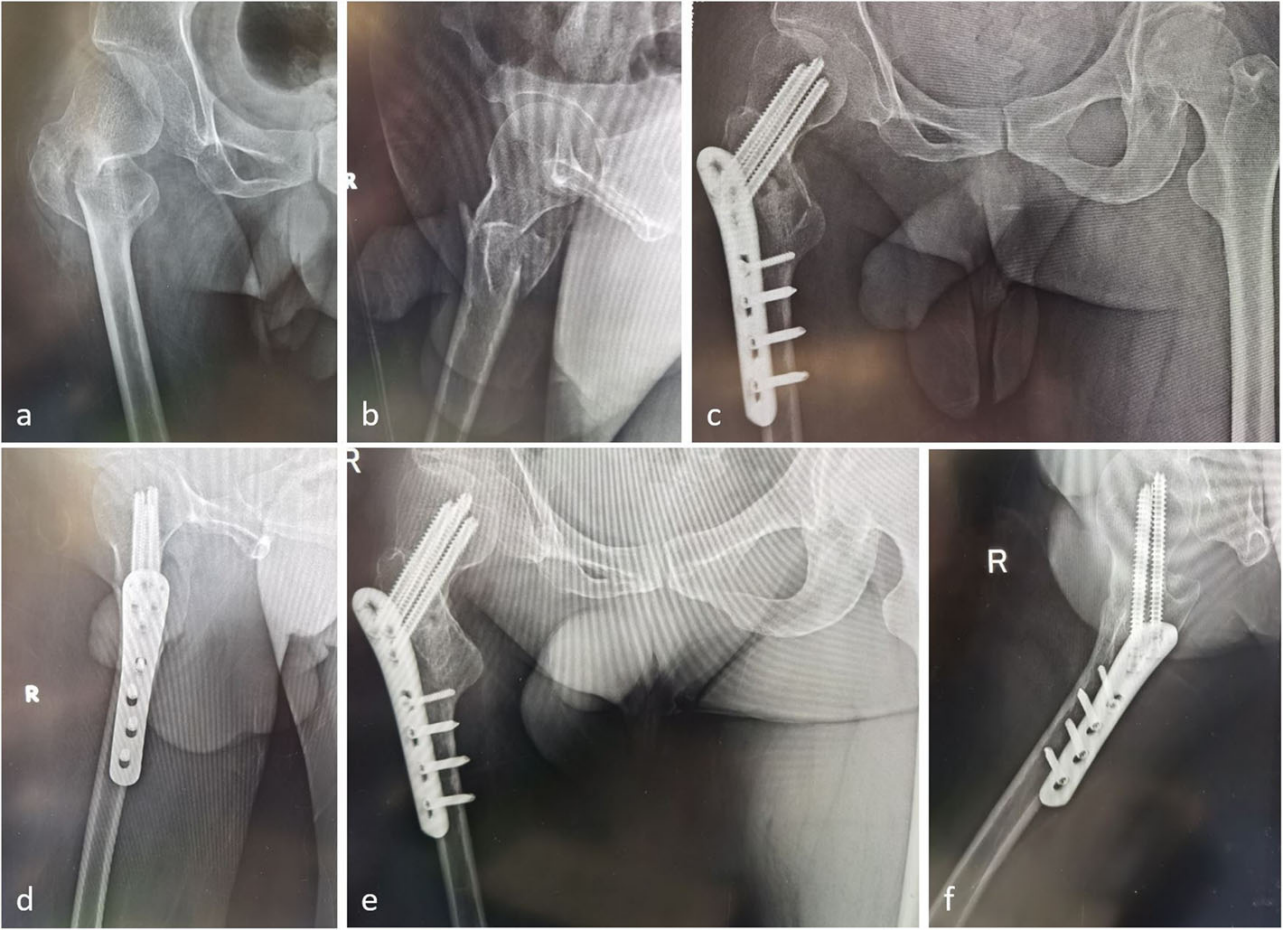
The patient was a 56-year-old male who accidently fell from a standing height causing right hip pain and limited activity, while past medical history indicated poliomyelitis sequela that had been present for decades. A preoperative anteroposterior X-ray (**a**) and a lateral X-ray (**b**) showed a right femoral intertrochanteric fracture. Postoperative X-ray radiographs of the anteroposterior (**c**) and lateral view (**d**) 7 days after surgery. X-ray radiographs of the anteroposterior (**e**) and lateral (**f**) 17 months after surgery indicated fracture union

There were no significant differences in HOOS scores between pre-injury and the last follow-up (*p* > 0.05, Table 1) among all 37 patients. No statistical differences were found in the SF-36 scores between pre-injury and the last follow-up either (*p* > 0.05, Table 2). The typical cases are shown in Figs. 1 and 2.

**Table 1:** Comparison of hip HOOS scores between pre-injury and the last follow-up in 37 patients with intertrochanteric fractures and preexisting proximal femur deformities (points, *x* ± *s*)

Characteristics	Pre-injury	Last follow-up	*t*	*p*
Pain	80.7 ± 12.4	78.7 ± 12.7	0.680	0.498
Symptoms	60.4 ± 20.0	58.4 ± 16.1	0.470	0.640
Activity limitations-daily living	56.8 ± 13.3	52.6 ± 14.7	1.299	0.198
Sport and recreation function	57.4 ± 12.1	52.9 ± 13.5	1.513	0.135
Quality of life	64.9 ± 16.0	61.9 ± 15.5	0.800	0.426

**Table 2:** Evaluation for the patients’ quality of life between pre-injury and the last follow-up after surgery (*x* ± *s*, *n* = 37)

Characteristics	Pre-injury	Last follow-up	*t*	*p*
Physical function	88.2 ± 12.4	83.9 ± 9.2	1.870	0.065
Role-physical	64.2 ± 21.7	60.1 ± 20.8	1.168	0.247
Bodily pain	71.3 ± 14.6	66.6 ± 16.7	1.291	0.201
General health	74.6 ± 11.4	73.6 ± 9.7	0.408	0.685
Vitality	72.7 ± 12.2	71.1 ± 11.7	0.582	0.562
Social functioning	72.3 ± 14.8	66.6 ± 14.5	1.691	0.095
Role-emotional	73.9 ± 24.7	65.3 ± 22.7	1.551	0.125
Mental health	63.7 ± 10.1	60.2 ± 8.2	1.614	0.111
Reported health transition	54.7 ± 11.5	52.0 ± 17.1	0.198	0.427

Two patients experienced postoperative pain in the proximal lateral thigh during hip flexion, which was probably due to the implant stimulation of the fascia. These symptoms disappeared after the fracture healed and internal fixation was removed. Other patients did not complain of similar types of pain after surgery.

## Discussion

Poor axial alignment is not the only manifestation of fracture reduction with preexisting proximal femoral deformity. Rotation abnormalities and coxa vara deformity also lead to complex and variable pathological changes of the proximal femur [14], such as a small and shortening femur, alternation in anteversion and neck shaft angle, rotation or hyperostosis of the greater trochanter, and abnormal femoral bone quality. The severity and extent of deformities depends on the type of primary disease and previous surgical history, which have a significant impact on surgical strategy, internal fixation selection, and placement. Therefore, compared with femoral intertrochanteric fractures without preexisting deformities, the management of patients with proximal femoral deformity is difficult to be managed due to the following reasons: 1) it is difficult to apply intramedullary fixation when the proximal femur is anatomically deformed; 2) the fracture site needs to be fully exposed and reduction needs to be performed based on the original anatomical structure, and minimally invasive techniques may not be an optimal option; 3) early weight bearing cannot be postoperatively fulfilled.

The advantages of using locking plates have been reported along with its wide clinical applications [15, 16]. Some authors [17, 18] have described that locking compression plates meet the biomechanical requirements for internal fixation of intertrochanteric fractures while providing many advantages, such as greater postoperative stability, lower incidence of complications and a broader spectrum of indications. Locking compression bone plates effectively improve postoperative quality of life of patients and are especially suitable for elderly patients with unstable intertrochanteric fractures and osteoporosis.

### Characteristics of femoral intertrochanteric fracture patients with preexisting deformity of the proximal femur

The AO/OTA classification of the 37 patients showed that 68% of the patients had an extra articular intertrochanteric fracture, including 20 cases of type 31-A3.1 and 5 cases of type 31-A3.2, whereas the other 32% of the patients suffered comminuted extra articular pertrochanteric fractures, including 6 cases of type 31-A2.1 and 6 case of type 31-A2.2. None of the 37 fractures were extremely comminuted, and the type 3 of the AO/OTA subgroup was not presented. Radiographic data analysis indicated that all fractures showed various degrees of deformity of the coxa vara. Previous studies [19, 20] have shown that the hip joint loaded approximately 3 to 5 times of the normal body weight due to the presence of a coxa vara deformity, leading to excessive loading on the tension side of the femur, which may cause local overload. Therefore, fractures of type A3 were the main pattern in our study due to the onset of preexisting coxa vara deformities before the injury and local overloading on the tension-side of the proximal femur due to additional weight-bearing. Meanwhile, the physical load increased on the tension side of the proximal femur when the tension-side suffered external loading until it exceeded the ultimate limit of bone toughness. Then the tension side would break first and the pressure side broke as soon as the force was conducted to the internal side. In addition, the affected limb was already disabled before the injury, therefore it would have been subconsciously protected during daily life. During the course of intertrochanteric fracture due to external force, the patient was expected to take protective actions to decrease the severity of injury.

In this study, we applied a locking compression plate to treat intertrochanteric fractures in 37 patients with proximal femoral deformity, and drew similar conclusions on the advantages of this option: 1) the shape of the plate can maximally match proximal femoral deformity without damaging the locking hole. 2) the locking compression plate did not affect the stability and grip strength of fracture fixation even if it could not completely and perfectly match proximal femoral deformity after reshaping. 3) due to the original disability in the affected limb, less daily stress stimulation and the onset of disused osteoporosis, screw loosening and cutting, which are likely to occur if regular plates are used. However, these problems may be solved by using a locking plate.

### Postoperative weight-bearing

For these patients, the opportune moment of postoperative weight-bearing should be taken seriously into account: (1) although the locking compression plate was very strong, it had to be reshaped to fit the particular fracture. This reshaping process might partially damage the plate and compromise its stiffness and toughness. If the internal fixation is subjected to weight-bearing overload before the fracture heals, the bone plate may break easily; (2) due to the disability of the affected limb before injury, the contralateral healthy limb became the main weight-bearing extremity when walking. Due to the relatively poor bone quality and quantity in the affected side, premature weight-bearing could result in failed internal fixation and screw loosening. Some authors [12, 21] have reported that coxa vara deformity often occurs after locking compression plate internal fixation due to severe osteoporosis and weight-bearing activity before the internal femoral cortex establishes adequate buttress function. For this reason, the standing timing and weight-bearing for such patients needs to be determined according to the type of fracture, osteoporosis, and fracture healing conditions.

### The possibility of performing osteotomy orthopedic surgery directly during the first stage

At present, most authors have focused on osteotomy orthopedic strategies for proximal femoral deformity without fracture and have emphasized on postoperative rehabilitation of the hip joint. To the best of our knowledge, there are only a few reports on management options for this particular type of fracture with preexisting deformities. In non-fracture cases, abnormal axial alignment and rotation are the main predicaments for orthopedic correction of proximal femoral deformity [22]. Some patients, such as those with congenital developmental dysplasia of the hip, are also affected by structural abnormalities, such as dysplasia, small bones, increased anteversion angle, and a backward great trochanter. The choice of osteotomy and internal fixation mainly depends on the degree of anatomical abnormalities [3]. Deng et al. [6] described that in patients with femoral deformities without associated fractures, THA, precise osteotomy, and correct choice of prostheses were key factors for femoral reconstruction and fixation. Kim et al. [23] reported that total hip arthroplasty with trochanteric ostectomy rendered favorable results for patients with deformity of the proximal femur. For these patients with more complicated pathological factors and osteotomy options, in addition to the deformities on the multi-planar surface, should be taken into account in order to maximally improve the function of the affected hip joint. 3D osteotomy of the proximal femur [24] can be used to treat most deformities because the original intertrochanteric or subtrochanteric bone is continuous. For patients with proximal femoral deformity who have suffered fractures, osteotomy cannot be achieved because the proximal femoral fracture line is likely to cross the osteotomy line. As a result, spreading, closing and rotation between the bone fragments after the osteotomy procedure cannot be performed following the original plan, which likely makes the case more complicated and increases the difficulty and risk of the surgery. Therefore, for fracture patients with preexisting proximal femoral deformity, it is not recommended for osteotomy and orthopedic treatment to be performed directly over the course of open reduction and internal fixation after fracture. Patients can be subject to osteotomy and orthopedic surgery if needed after the fracture has healed and the affected limb is able to bear weight-bearing activities.

### The surgical significance of fracture fixation and maintenance of the original skeletal deformity

In patients with femoral trochanter deformity, soft tissues around the hip joint are adapted to this deformed condition and a new level of homeostasis must be established before fracture. If the original condition of bone deformity is not maintained, the balance between soft tissues and the hip joint may be disequilibrated and unpredictable issues, such as pain, abnormal gait, and lifestyle changes, may emerge. Our aim was to enable the patients to recover with a painless hip joint instead of a movable but painful one. Therefore, it is believed that we have reached a consensus on the primary intention of the Harris Score Scale [25], in which the distribution score is more focused on postoperative pain and joint function changes instead of joint activity. This was further confirmed by the finding that no significant differences were found among the 37 patients based on the HOOS score of the hip joint between pre-injury and last follow-up after fracture fixation. These patients were disabled before surgery, and their contribution to the family and society had certainly been compromised. By maintaining the original skeletal malformation in our fixation surgery, we could address family and social issues raised by fractures to provide the lowest economic burden. At the end of the follow-up period, no significant differences were found in the 8 dimensions of quality of life between pre-injury and after surgery, which indicated that after the fractures had healed, the quality of life of the patient was restored to pre-injury levels. In terms of the quality of life of patients, we achieved our primary objectives of treatment.

There are some limitations of our study that should be clarified. The sample size of our retrospective study was relatively small, which may enhance the probability of bias in the statistical results. Thus, further prospective studies with larger sample sizes are required to validate our findings. When applying the locking compression plates for the treatment of these patients, flexibility for reshaping the locking compression plate was limited since the procedure must be performed without damaging the thread of the locking nail. For this reason, some locking screws could not be screwed in at the designated direction and sometimes the screws had to be screwed in another direction or position. Instead of being drilled and screwed in the designated locking sleeve direction, the screws must be drilled and screwed into the fixed direction as required and sometimes cold-welding must be simultaneously performed to achieve stability. The rigidity and toughness of the compression plates changed after reshaping. Therefore, since patients could not fulfill early weight-bearing after surgery and take care of themselves during rehabilitation, the resulting family and social burden was increased to some degree.

## Conclusion

In summary, we believe that fracture reduction and fixation by lock compression plate is a simple and effective method that is suitable to be used to treat patients with preexisting proximal femoral deformities. This study also has some limitations, including it being a retrospective study rather than prospective study, the inclusion of multiple types of proximal femoral deformities, with an extremely limited sample size of each type of deformity. Further in-depth analyses needs to be conducted when a larger sample size becomes available.

## Data Availability

The data and materials are available from the medical records department of Xi’an Honghui Hospital.

## Abbreviations

HOOS: The hip disability and osteoarthritis outcome score
SF-36: The short form 36 Health Survey Questionnaire
CPM: Continuous passive motion
